# Effect of Post-Processing Heat Treatment Temperature on Microstructural Evolution and Mechanical Properties of the Ti-6Al-2Sn-4Zr-2Mo Alloy Fabricated by Laser Powder Bed Fusion

**DOI:** 10.3390/mi17010016

**Published:** 2025-12-24

**Authors:** Kanghyun Park, Yunjong Jung, Seongjin Im, Kangjin Lee, Mincheol Kwon, Soonjik Hong, Jongun Moon, Junmo Seong, Jinman Park, Gian Song

**Affiliations:** 1 Center for Advanced Materials and Parts of Powder, Division of Advanced Materials Engineering and Institute for Rare Metals, Kongju National University, 1223–24, Cheonan-daero, Seobuk-gu, Cheonan-si 31080, Chungnam, Republic of Koreayjjung@smail.kongju.ac.kr (Y.J.); sjlim@smail.kongju.ac.kr (S.I.); kjlee@smail.kongju.ac.kr (K.L.); mck08150@smail.kongju.ac.kr (M.K.);; 2 Global Technology Research (GTR), Samsung Electronics Co., Ltd., 129 Samsung-ro, Yeongtong-gu, Suwon-si 16677, Gyeonggi-do, Republic of Korea

**Keywords:** Ti-6Al-2Sn-4Zr-2Mo, mechanical properties, heat treatment, laser powder bed fusion, post-processing

## Abstract

In this study, the influence of post-processing heat treatment on microstructure and mechanical properties of Ti-6Al-2Sn-4Zr-2Mo (Ti-6242) alloy fabricated by laser powder bed fusion (L-PBF) was investigated. The mechanical properties of the as-built and heat-treated samples with various temperatures (600–850 °C) were evaluated using a tensile test at room temperature. After heat treatments, both yield strength (YS) and ultimate tensile strength (UTS) gradually decreased, while the tensile elongation tended to increase as the heat treatment temperature increased. These variations were closely related to the microstructural evolution caused by heat treatment. Specifically, the decomposition of α′ martensite into the α + β lamellar structure and subsequent coarsening were promoted with increasing temperature, leading to stress relief and improved dislocation storage capability, which resulted in the variation in mechanical properties. Notably, although the mechanical strength was reduced after heat treatment with increasing temperatures, the lowest yield strength and ultimate tensile strength were measured as 1086.4 ± 16.5 and 1135.0 ± 15.0 MPa, respectively, which are comparable to or higher than those of conventionally processed Ti-6242. As a result, the post-processing heat treatment could be an effective approach to achieve desirable performance for targeted applications.

## 1. Introduction

Ti-6Al-2Sn-4Zr-2Mo (Ti-6242) is a near-α titanium alloy that exhibits an outstanding combination of tensile strength, creep resistance, and oxidation resistance up to 538 °C [1]. These excellent high-temperature properties render Ti-6242 highly suitable for critical components in the aerospace and power generation industries, such as compressor disks, turbine blades, and compressor casings, where long-term exposure to elevated temperatures and high stresses is required [2]. Such performance is achieved through a balanced alloy design strategy (i.e., Al and Mo act as α and β stabilizers, respectively, whereas Sn and Zr contribute to strengthening the α phase by the solid solution strengthening effect) and complex thermo-mechanical processing, such as rolling, forging, and heat treatment [3]. However, the intrinsic properties of Ti-6242 alloy, such as high strength, low thermal conductivity and elastic modulus, make it difficult to machine a desirable shape [4].

The additive manufacturing (AM) technique has attracted considerable attention due to its specialty in products with desired shapes and outstanding mechanical properties [5,6,7]. In particular, the laser powder bed fusion (L-PBF) method has emerged as a promising alternative for the fabrication of near-net-shape components with complex geometries [8,9]. The high-energy laser selectively melts successive powder layers, producing extremely high cooling rates (10^3^–10^6^ K/s) and steep thermal gradients [10]. Such conditions result in microstructures markedly different from those of conventionally processed alloys, including acicular martensitic α′ and pronounced textures [11,12,13]. While these features can lead to high strength in the as-built condition, they are often accompanied by high residual stresses [14], anisotropic mechanical properties caused by columnar grains [15], and reduced high-temperature performance [16], which limit the direct use of as-built L-PBF Ti-6242 components for demanding service environments.

Post-processing heat treatments have been employed as a critical step for tailoring the microstructure and properties of various L-PBF Ti alloys. The heat treatment parameters, such as temperature, time, and cooling rate, govern the important microstructural features, i.e., α/β phase fractions, grain size, and α lamellar morphology, thereby controlling tensile strength, ductility, and creep resistance [2,3]. For instance, many studies on L-PBF Ti6Al4V alloys have reported that appropriate heat treatments decompose the α′ martensite into α + β lamellar, changing the α phase morphology, such as grain shape, size, and colony size, thereby achieving a good balance of the strength and ductility [13,17,18,19,20]. In contrast, while extensive work has been conducted on the heat treatment of wrought Ti-6242, systematic investigations focused on L-PBF Ti-6242 remain limited [21,22,23,24]. Specifically, previous studies largely focused on higher sub-transus temperatures (approximately 850–1000 °C) and employed long dwell times or multistep heat treatment schedules [21,24,25]. However, there is a lack of systematic investigation into the intermediate sub-transus range (600–850 °C) using short, single-step treatments, although this range is critical because it governs the transition from the hard α′ martensite to a ductile α + β microstructure. Furthermore, identifying effective heat treatments at lower temperatures and shorter dwell times is highly relevant for reducing the overall energy and cost of post-processing. Thus, the heat treatment at intermediate temperatures (600–850 °C for 2 h) would provide an additional understanding between microstructure and mechanical properties, and further, a reduction in component production cost.

In this study, the objective is to investigate the microstructural evolution and mechanical properties through one-step post-processing heat treatment and compare the mechanical properties with conventional Ti-6242 alloy to identify the optimal heat treatment condition and the possibility for applications as structural materials in various industries. Specifically, Ti-6242 alloys fabricated by L-PBF were subjected to post-processing heat treatments at 600–850 °C for 2 h. The results demonstrate that the optimized heat treatment can significantly simplify the processing route while achieving mechanical properties comparable to, or even superior to, those of conventionally wrought Ti-6242. Importantly, the present findings highlight that post-processing heat treatment provides a practical pathway to achieve the target performance, thereby supporting the feasibility of implementing L-PBF for Ti-6242 components in real applications.

## 2. Materials and Methods

**Figure 1:**
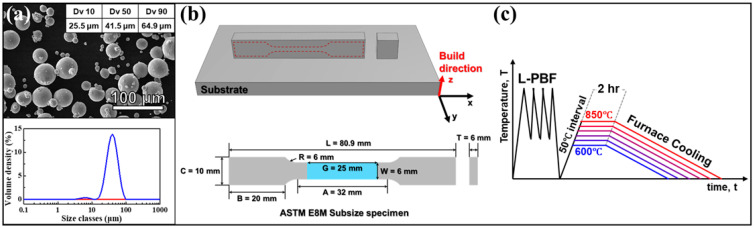
(**a**) SEM image of Ti-6242 powder and result of particle size analysis, the particle size distribution is shown by the blue curve, and the red line indicates the 0% baseline. (**b**) A schematic of L-PBF-processed specimen for the tensile test and microstructure analysis; (**c**) temperature–time profile of the Ti-6242 alloy during the L-PBF process followed by heat treatment.

The Ti-6Al-2Mo-4Zr-2Sn (wt.%) powder with spherical morphology (Figure 1a) was provided by ECKART (Hartenstein, Germany), manufactured by the electrode induction melting gas atomization (EIGA) method. The average powder size was measured, ranging from 25.5 to 64.9 μm, with a mean of 41.5 μm and an approximately spherical morphology, as shown in Figure 1a. This was analyzed using a particle size analyzer (Mastersizer 3000E, Malvern Panalytical, Malvern, UK) with wet sieving methods using ethanol. In addition, the chemical composition of the feedstock powder is analyzed using an SEM-EDS on five different particles. The average compositions are shown in Table 1.

The bulk specimens of cubic and dog-bone shape for microstructure analysis and mechanical tests were fabricated by laser powder bed fusion (L-PBF) using the Ti-6242 alloy powder. The L-PBF samples were fabricated using a laser power of 280 W, a scanning speed of 1200 mm/s, a hatch spacing of 140 µm, and a layer thickness of 30 µm. The build orientation of all samples was aligned along the Z-axis. In addition, the as-built L-PBF samples exhibited an average density of 4.5305 ± 0.0057 g/cm^3^, corresponding to a relative density of 99.79 ± 0.12%, measured by the Archimedes method. Heat treatment was conducted on as-printed samples for 2 h at a temperature range of 600–850 °C, respectively. The cubic specimens for microstructure characterization and phase identification were mechanically ground using the SiC paper with 400–4000 grit, followed by polishing with 0.02 μm colloidal silica suspension. To analyze the microstructure, the samples were etched using Kroll’s reagent composed of 3 mL of hydrofluoric acid (HF), 6 mL of nitric acid (HNO_3_), and 100 mL of distilled water (approximately 2.8 vol% HF, 5.5 vol% HNO_3_, and 91.7 vol% H_2_O).

To analyze the microstructural evolution with different heat treatment conditions, field-emission scanning electron microscopy (FE-SEM, TESCAN MIRA II) with a backscatter electron (BSE) detector was conducted. The crystallographic characterization was carried out by an X-ray diffractometer, D8 ADVANCE (Bruker, Billerica, MA, USA) with Cu Kα (λ = 1.5406 Å) on bulk cubic samples with a 0.02° step size and a scan speed of 1°/min from 30° to 80° of the 2θ range. Room temperature mechanical properties of as-built and heat-treated Ti-6242 samples were evaluated by uniaxial tensile tests on a dog-bone-shape specimen with a gauge length of 25 mm and a rectangular cross-section of 3 mm × 5 mm under a strain rate of 10^−3^ s^−1^. During the tensile test, an extensometer with a gauge length of 25 mm was utilized for precise measurement of the tensile elongation.

## 3. Results and Discussion

### 3.1. Mechanical Properties Evaluated by Tensile Tests

**Figure 2:**
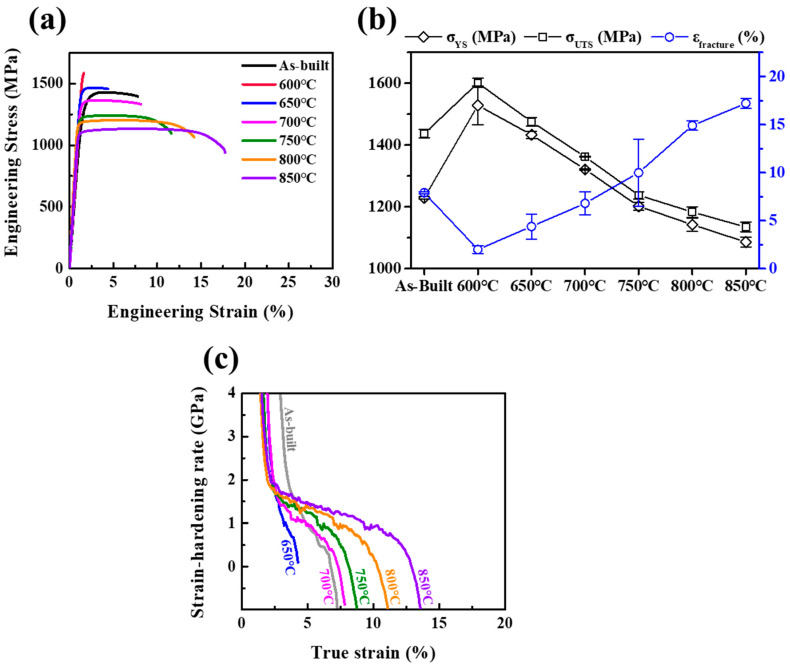
Tensile behaviors of as-built and heat-treated Ti-6242 alloys. (**a**) Engineering tensile stress–strain curves, (**b**) summarized mechanical properties, and (**c**) strain-hardening rate curves of corresponding tensile curves of the alloy.

Figure 2a shows representative engineering stress–strain curves of the as-built and heat-treated Ti-6242 samples under room temperature tension. The as-built sample exhibits YS of 1229.3 ± 4.7 MPa, UTS of 1437.2 ± 13.0 MPa, and 7.9 ± 0.1% of elongation. The samples subjected to heat treatments in the range of 600–850 °C at 50 °C intervals show a systematic variation in mechanical properties as the temperature increased. Figure 2b displays the variation in the YS, UTS, and fracture elongation as a function of heat treatment temperatures, and the as-built sample’s values are included for comparison. The heat treatment at 600 °C induces a substantial increase in UTS to 1601.4 ± 15.1 MPa, with insufficient elongation. As the heat treatment temperature increases, YS and UTS gradually decrease while tensile elongation increases. These trends are maintained to 850 °C, showing decreased YS and UTS to 1086.4 ± 16.5 and 1135.0 ± 15.0 MPa and increased elongation to 17.2 ± 0.5%. The specific mechanical properties, such as yield strength (YS), ultimate tensile strength (UTS), and tensile elongation to the fracture, are listed in Table 2. Based on these results, it is believed that heat treatment temperature significantly affects the mechanical properties of the L-PBF Ti-6242 samples.

Moreover, the strain-hardening rate curves of the as-built and heat-treated Ti-6242 samples are exhibited as a function of true strain, as depicted in Figure 2c. Note that the sample heat-treated at 600 °C is omitted due to the insufficient elongation. As heat treatment temperature increases from 650 to 850 °C, the strain-hardening rate shows a gradual increase, and the highest strain-hardening behavior is reached at 850 °C. In general, it has been reported that the improved strain-hardening effectively suppresses strain localization and delays the onset of necking, resulting in larger uniform elongation [26]. Therefore, the improvement in ductility after heat treatment could be closely associated with increased strain hardening.

**Table 2:** Mechanical properties of as-built and heat-treated Ti-6242 samples and conventional Ti-6242 alloy [27].

Heat Treatment Condition	0.2% Offset Yield Strength (MPa)	Ultimate Tensile Strength (MPa)	Fracture Elongation (%)
As-built	1229.3 ± 4.7	1437.2 ± 13.0	7.9 ± 0.1
600 °C	1528.8 ± 62.6	1601.4 ± 15.1	2.0 ± 0.4
650 °C	1433.7 ± 11.9	1475.8 ± 13.9	4.4 ± 1.3
700 °C	1321.4 ± 2.9	1362.5 ± 1.9	6.8 ± 1.2
750 °C	1202.5 ± 13.1	1238.1 ± 11.7	10.0 ± 3.5
800 °C	1142.5 ± 21.9	1183.6 ± 16.9	14.9 ± 0.5
850 °C	1086.4 ± 16.5	1135.0 ± 15.0	17.2 ± 0.5
Timet-6242 STA 970 °C/1 h + 595 °C/8 h [27]	955	1045	18

### 3.2. Phase Identification Using X-Ray Diffraction

From the results of the mechanical tests, the mechanical properties were largely dependent on the heat treatment temperature. Since the constitutive phases and microstructure are closely related to the mechanical properties, we representatively selected the three different samples (600, 700, and 800 °C) showing distinct strength–ductility trade-off to investigate the phase and microstructural evolutions.

**Figure 3:**
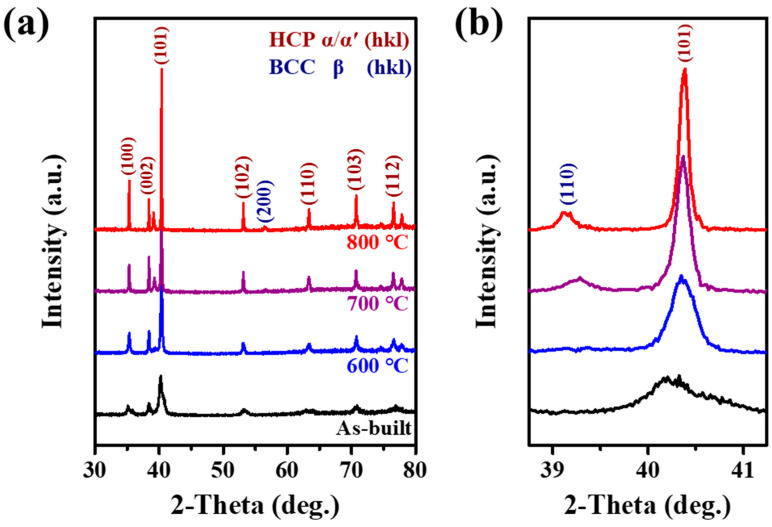
XRD patterns of as-built and aged Ti-6242 alloys with a range of 2θ for 30~80° (**a**), with an upscaled range of 38~41° (**b**).

Figure 3 presents the XRD patterns of the as-built and heat-treated samples at 600, 700, and 800 °C. As shown in Figure 3a, the as-built sample exhibits the broad HCP diffraction peaks without any peaks associated with additional phases. After heat treatment at 600 °C, the single HCP phase is still retained without the formation of other phases, whereas the peak width becomes narrower compared to that of the as-built sample. In contrast, heat treatments at 700 and 800 °C lead to the occurrence of BCC peaks in the vicinity of 39°, where the peak intensity gradually increases with increasing temperature, indicating an increase in the volume fraction of the BCC phase (Figure 3b). These results suggest that heat treatment temperature plays an important role in the variation in constitutive phases.

It is well known that the peak broadening is related to the residual stress and lattice distortions [28]. The residual stress is generated during the AM process due to rapid and repeated cooling/melting [29]. Moreover, rapid solidification/cooling can suppress the elemental diffusion and thus lead to martensitic transformation from β to α′ in near-α Ti alloys [22,24,30]. In other words, the α′ phase is supersaturated with BCC elements, resulting in distortion in the HCP lattice structure [23]. Therefore, it is believed that the peak broadening in the as-built sample is closely related to the presence of residual stress and martensitic α′ phase formed during the AM process.

On the other hand, the variation in peak width and the formation of the BCC phase were observed after heat treatment. From various studies on the heat treatment of the AM samples, it has been reported that the residual stress can be relieved by thermal activation [31,32,33]. In addition, the martensitic α′ phase could decompose into two equilibrium phases, HCP-structured α phase and BCC-structured β phase during heat treatment at temperatures ranging from 750 to 800 °C [34]. Considering these results, the reduction in HCP peak width in the heat-treated sample at 600–800 °C could be due to the relief of the stress. Furthermore, the formation of the BCC phase at higher temperatures (700 and 800 °C) is attributed to the decomposition of the martensite.

### 3.3. Microstructural Analysis

Despite the presence of hard martensite and residual stress in the as-built sample, it exhibited a relatively desirable elongation (7.9 ± 0.1%) with high strength (1229.3 ± 4.7 and 1437.2 ± 13.0 MPa for YS and UTS, respectively). In contrast, although the residual stress was relieved by heat treatment at 600 °C, a large increase in strength and insufficient elongation was observed (Figure 2b). These phenomena cannot be fully explained by XRD results. Thus, an additional microstructural analysis was conducted to establish the underlying mechanism.

**Figure 4:**
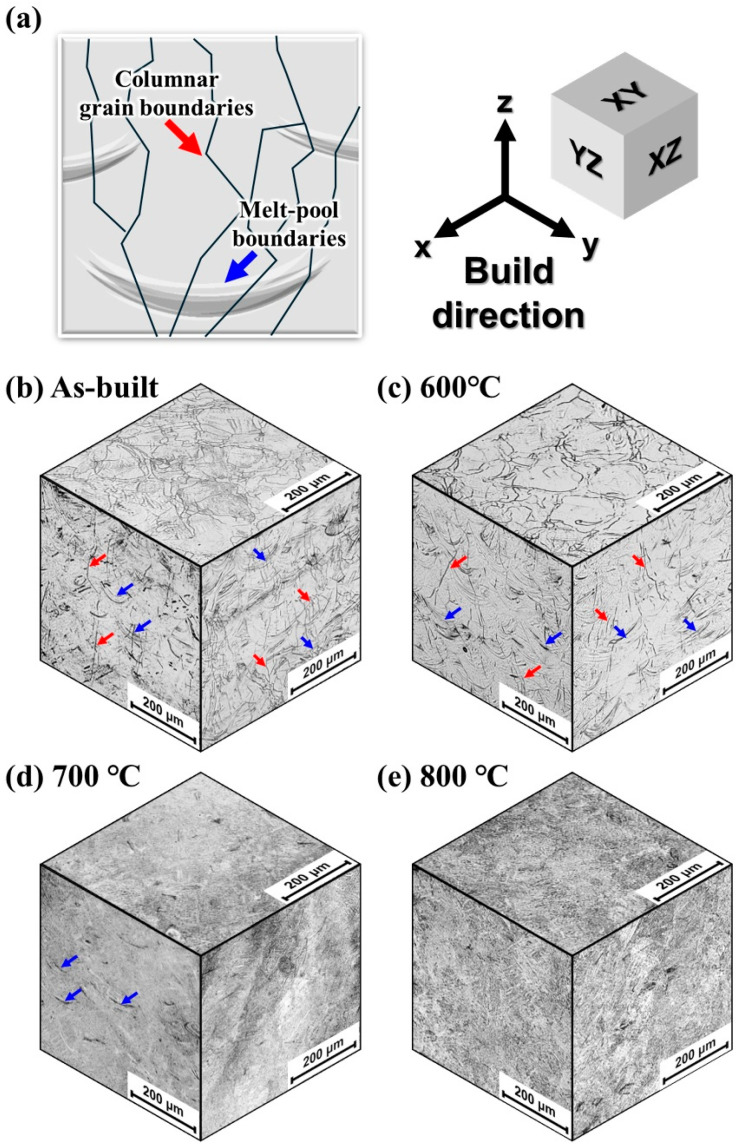
(**a**) Schematic illustration of the microstructure of the L-PBF sample on the XZ and YZ planes; (**b**) as-built microstructure; (**c**–**e**) microstructures after heat treatment at 600, 700, and 800 °C, respectively.

Figure 4a illustrates the schematic of the microstructure of the L-PBF manufactured alloy on the XZ and YZ planes, which mainly consists of a columnar structure (red arrows) and melt-pool boundaries (blue arrows). Figure 4b shows the microstructures of the as-built sample. The as-built sample clearly exhibits distinct equiaxed grain shape on the XY plane, whereas the columnar structure and melt-pool boundaries are revealed along the building (Z) direction on the lateral surfaces. These features are observed in the heat-treated sample at 600 °C (Figure 4c). However, with increasing temperatures (700 and 800 °C), the columnar and melt-pool boundaries gradually disappeared (Figure 4d,e), which could be due to the homogenization of chemical segregation [35].

**Figure 5:**
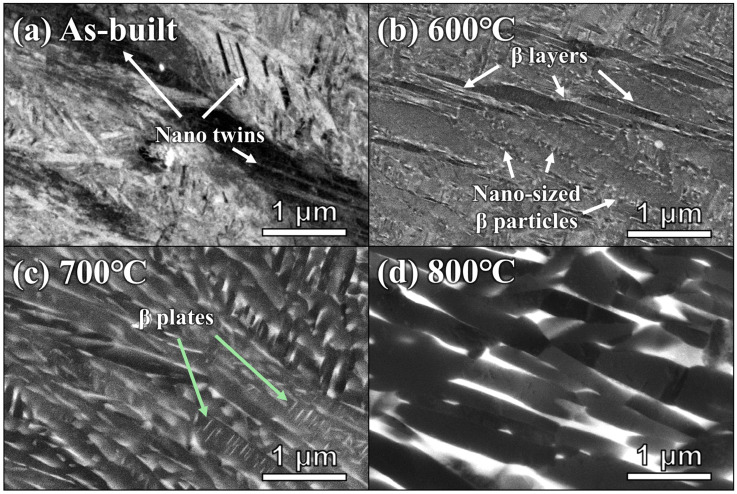
SEM-BSE images of the as-built and heat-treated Ti-6242 alloys at 600, 700, and 800 °C: (**a**) as-built, (**b**) 600 °C, (**c**) 700 °C, and (**d**) 800 °C.

Figure 5a–d display the SEM images of the as-built and heat-treated Ti-6242 samples measured on the Y-Z plane, which clearly reveal the microstructural evolution with increasing temperatures. The as-built sample exhibits fine acicular α′ martensite, which has often been observed in the as-built samples of the Ti alloys with low β stabilizer contents [23,30]. In addition, several band structures are formed within the α′ martensite, implying the presence of nanotwins [21,22,23]. After heat treatment at 600 °C, α′ martensite is partially decomposed into α and β phases, while retaining acicular morphology (Figure 5b). Specifically, α phases show a lamellar shape with a wide range of thickness for 10–500 nm and dark contrast, while nano-sized 10–50 nm β precipitates or layers with bright contrast are located among the α lamellar. Moreover, the nano-sized precipitates could be a reason why they were not detected in the XRD patterns [36]. At 700 °C, martensitic decomposition occurs more actively, and the α + β lamellar structure becomes more pronounced. Moreover, both phase fraction and size of β phase considerably increase compared to 600 °C. In addition, thin β plates were formed densely within the α lamellar, as marked by the green arrow in Figure 5c. It is considered that the thin β plates formed at prior twin boundaries within α′ martensite during martensite decomposition [37]. With an increase in temperature to 800 °C, α′ martensite is completely decomposed to α and β phases with basket-weave morphology (Figure 5d). The α lamellar becomes thicker than that of the heat-treated sample at 700 °C, exhibiting a width range of 200–1000 nm and accompanying the coarsening of the β layer (40–180 nm) surrounding the α plates.

**Figure 6:**
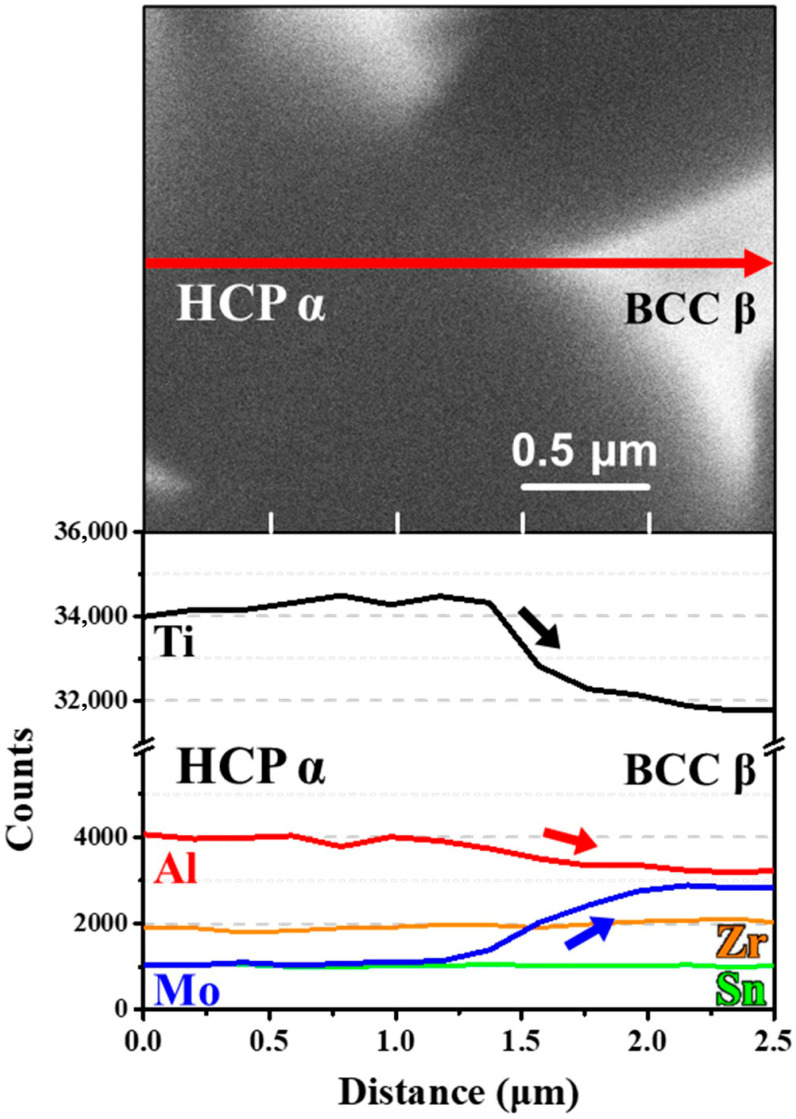
Compositional analysis using SEM-line scanning for the sample heat-treated at 800 °C.

To clarify the constitutive phases, we additionally conducted line scanning. Note that because the bright phase was very small, it was difficult to detect the compositional distribution except for the sample heat-treated at 800 °C. In this sense, line scanning was performed for the sample heat-treated at 800 °C. Figure 6 shows the line scanning results, which clearly exhibit the different elemental distribution in dark and bright contrast regions. For instance, the dark phase contains Ti and Al elements, whereas the bright phase is enriched in Mo elements with depletion of Ti and Al elements. Based on these results, the dark phase is considered to be the α phase, whereas the bright phase is identified as the β phase, as reported in the previous studies [21,25].

Despite the presence of residual stress induced by the repetitive rapid heating and cooling cycles during the AM process, the as-built sample exhibited a relatively high elongation (Figure 2). The SEM observation of the as-built sample showed acicular α′ martensite and dense nanotwins within the martensite. The nanotwins within α′ martensite have been reported in L-PBF Ti-6Al-4V [23], Ti-6Al-2Zr-1Mo-1V [30], and Ti-6242 [21] alloys. Although α′ martensite is well known as a metastable phase with hard and brittle qualities [18], Fan et al. [22] reported that nanotwins can provide additional space for dislocation accumulation, thereby enhancing both strength and ductility. Thus, the mechanical properties of the as-built sample could result from the synergy of a hard α′ martensite and the formation of nanotwins.

The sample heat-treated at 600 °C exhibited a UTS of 1601.4 ± 15.1 MPa with insufficient elongation. This is contrary to the typical deformation behavior that shows residual stress relief when the aging and/or annealing treatments are applied to the AMed materials [19,20]. It could originate from the presence of diffusion sites. Specifically, it has been reported that dense martensite and twin boundaries in the as-built sample act as a favorable region for diffusion during the martensitic decomposition, thereby facilitating β precipitation [21,22,37]. Indeed, the SEM observations clearly showed the presence of the nano-sized β precipitates and layers inside martensite and along the boundaries (Figure 5b). In other words, the formation of nano-sized β precipitates around prior twin/martensite boundaries after heat treatment could promote an increase in strength and a decrease in elongation by impeding dislocation motion [21,38]. Thus, these results reflect that the β precipitates and layers contribute to the variation in mechanical properties, such as strength and ductility.

Meanwhile, the heat treatment at 700 °C promoted the decomposition of α′ martensite and growth of β precipitates, which shows a continuous layer between the α grains. These trends were more pronounced at 800 °C, showing the basket-weave morphology with a thicker α phase width and a coarsened β phase. Moreover, as shown in the XRD pattern, the peak width of the α phase became narrow and the β peak appeared with increasing temperature (Figure 3). These indicate that the heat treatment at a higher temperature range of 700–800 °C relieved the residual stress and alleviated the supersaturation of solute elements, resulting in the improvement of dislocation mobility and the softening of the α phase [25]. In addition, coarsening of lamellar increases the mean free distance for dislocation slip [39], which could enhance the plastic deformation of L-PBF Ti-6242. Indeed, the improved strain-hardening rate with increasing temperature can support these, as shown in Figure 1c. As a result, it can be concluded that the reduction in strength and increase in elongation with increasing temperature are attributed to the decomposition of α′ martensite and growth of β precipitates.

### 3.4. Fracture Surface Analysis

From the phase and microstructural evolution, it was demonstrated that the mechanical properties were associated with the martensitic decomposition and growth. To further clarify the correlation between the microstructure and mechanical properties, fractography analysis was additionally conducted.

**Figure 7:**
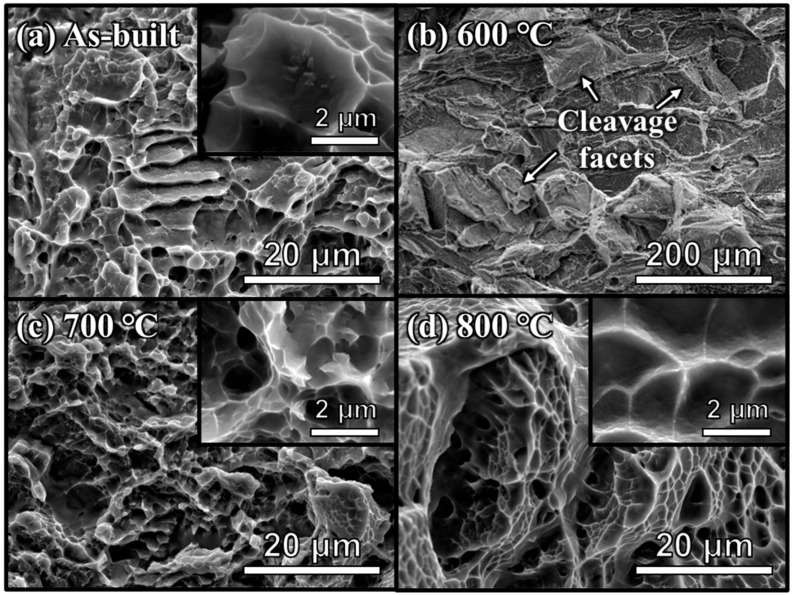
SEM images of fracture surface of (**a**) as-built and heat-treated samples at (**b**) 600 °C, (**c**) 700 °C, and (**d**) 800 °C. The images on the upper right show a dimple shape in high magnification.

Figure 7 presents the fracture morphologies of the as-built and heat-treated samples (600, 700, and 800 °C) after the tensile test. No noticeable printing defects, such as unmelted powders or pores, are observed in all samples. Figure 7a shows the fracture surface of the as-built sample, which consists of a mixture of large dimples (several-micron-sized) and fine dimples (sub-micron-sized), indicating a ductile-type fracture characteristic [21,22,30]. On the other hand, Figure 7b–d display the fractography of the heat-treated samples. Specifically, the typical cleavage facets are revealed after heat treatment at 600 °C, indicating a brittle fracture mode. With increasing temperature to 700 °C, a mixed morphology of small and large dimples is observed, similar to the as-built sample. At 800 °C, an increase in the size of dimples as well as the presence of rounder and smoother tear ridges compared to the other samples are observed, as shown in Figure 7c,d.

The brittle surface observed in the sample heat-treated at 600 °C could be due to an increase in local stress caused by partial decomposition of α′ martensite and the formation of β precipitates and layers [22,37]. In contrast, the dimples are well known as characteristics of ductile fracture. The relationship between dimple size and ductility has been investigated in various alloys [40,41]. Specifically, larger grain sizes lead to higher tensile elongation and correspondingly larger dimple diameters. Indeed, the grains gradually coarsened with an increasing heat treatment temperature, as shown in Figure 5. Similarly, relatively small-sized dimples were observed in the sample heat-treated at 700 °C, and a further increase in temperature to 800 °C led to the coarsened dimples (Figure 7c,d). These results suggest that the decomposition of α′ martensite into the α + β lamellar structure and their coarsening by heat treatment result in a transition from brittle to ductile nature.

### 3.5. Comparison with Previously Reported Ti-6242 Alloys

**Figure 8:**
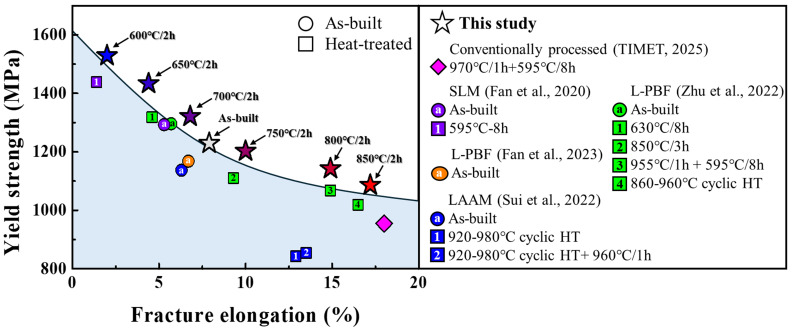
Comparison of yield strength and tensile elongation between the Ti-6242 of the present study, and reported data with conventional processed (Timet-6242 [27]), as-built, and heat-treated conditions of other AM processes (SLM [22], L-PBF [21,23], LAAM [25]).

Post-processing heat treatment is widely recognized as essential for achieving a balanced strength–ductility response in additively manufactured titanium alloys [17,18,25,42]. Indeed, this can be applied to the present L-PBF Ti-6242. Figure 8 shows the comparison of the yield strength and fracture elongation for our Ti-6242 samples (heat-treated below the β-transus at 600–850 °C for 2 h) with conventionally processed counterparts [27] and previously reported AM materials in the as-built and heat-treated conditions [21,22,23,25]. The alloys treated in the sub-transus range plot on or near the upper envelope of the literature strength–ductility scatter, consistently exhibiting superior combinations of yield strength and ductility. In particular, relative to SLM + direct aging [22], LAAM + cyclic heat treatment near the β-transus [25], and L-PBF + direct aging/STA/cyclic heat treatment [21], our single-step schedule delivers a comparably higher strength–ductility balance. These findings demonstrate that a short, single sub-transus treatment applied directly after L-PBF can achieve competitive performance without HIP or multistep STA cycles. Consequently, this route offers a simplified and cost-effective pathway to produce Ti-6242 with properties suitable for high-temperature aerospace components such as turbine blades.

## 4. Conclusions

In this study, we applied various temperatures of post-processing heat treatment on a Ti-6242 alloy manufactured by the laser powder bed fusion (L-PBF) process. The effect of various heat treatment temperatures on microstructure and mechanical properties was studied. The conclusions can be summarized as follows:

1. The as-built sample showed increased strength and relatively reduced elongation (1229.3 ± 4.7, 1437.2 ± 13.0 MPa, and 7.9 ± 0.1% for YS, UTS, and elongation, respectively), compared with the conventional Ti-6242 alloy, as shown in Table 2. This is due to the presence of the hard α′ martensite and twins within the α′ martensite.
2. Heat treatment at 600 °C resulted in an insufficient elongation with a brittle surface, which was attributed to the formation of nano-sized β precipitates and layers caused by partial decomposition of α′ martensite.
3. Heat treatment at higher temperatures (700 and 800 °C) exhibited a distinct decomposition of α′ martensite into the α + β lamellar structure and their growth, which led to a decrease in strength and an increase in elongation, showing a ductile surface with dimples.
4. Compared with commercially processed Ti-6242 (casting and STA) and previously reported additively manufactured Ti-6242 alloys, the lowest yield strength and ultimate tensile strength are measured as 1086.4 ± 16.5 and 1135.0 ± 15.0 MPa, respectively, along with improved elongation in this study. These tensile properties are comparable to or higher than those of conventionally processed Ti-6242, indicating that appropriate post-processing heat treatments enable L-PBF Ti-6242 to reach performance levels suitable for practical applications.

## Figures and Tables

**Table 1:** SEM-EDS analysis on the Ti-6242 powder is conducted on five different particles, and their average and standard deviation are summarized.

	Elements (Wt. %)				
Powder	Ti	Al	Sn	Zr	Mo
Ti-6242	Bal.	5.1 ± 0.08	1.64 ± 0.13	3.52 ± 0.23	2.16 ± 1.29

## Data Availability

Data is contained within the article.
